# Diffuse pneumonitis from coronavirus HKU1 on checkpoint inhibitor therapy

**DOI:** 10.1136/jitc-2020-000898

**Published:** 2020-05-19

**Authors:** Michael T Serzan, Princy N Kumar, Michael B Atkins

**Affiliations:** 1 Oncology, Georgetown Lombardi Comprehensive Cancer Center, Washington, DC, USA; 2 Infectious Diseases, Georgetown University Medical Center, Washington, DC, USA

**Keywords:** melanoma, immunotherapy, immunomodulation, case reports

## Abstract

**Background:**

Immune checkpoint inhibitors (ICIs) can produce specific immune-related adverse events including pneumonitis. The impact of ICI therapy on the severity of acute coronavirus infection symptomatology warrants further exploration.

**Case presentation:**

We report a 65-year-old man diagnosed with stage IV melanoma who developed pulmonary and brain metastases and was treated with bilateral craniotomies followed by combined nivolumab and ipilimumab immunotherapy. He developed early-onset severe dyspnea associated with acute coronavirus HKU1 (non-COVID-19) infection, with diffuse pneumonitis evidenced by ground glass opacification on CT scan. He was treated with steroids leading to resolution of pneumonitis on repeat imaging, suggesting an exacerbated immune-mediated toxicity.

**Conclusion:**

We report the first case of a patient with melanoma with severe and reversible diffuse pneumonitis in association with coronavirus HKU1 following combined nivolumab and ipilimumab immunotherapy. Although we do not have data on the impact of ICI therapy on severe acute respiratory syndrome coronavirus 2 (SARS-CoV-2) symptomatology, a possible interaction should be considered when deciding on dosing in patients with possible SARS-CoV-2 exposure or when evaluating patients with presumed ICI-related pneumonitis during the COVID-19 pandemic.

## Background

Ipilimumab and nivolumab are recombinant human monoclonal antibodies which target cytotoxic T-lymphocyte-associated antigen-4 (CTLA-4) and programmed death-1 (PD-1) receptor, respectively. Immune checkpoint inhibitors (ICIs) enable the restoration of endogenous antitumor immunity and have revolutionized treatment of advanced melanoma among other malignancies.^1–3^ Blockade of immune checkpoints has been associated with immune-related adverse events (irAEs) resulting from excessive inflammation in various organs.^4^ Checkpoint inhibitor pneumonitis (CIP) is characterized by dyspnea and/or other respiratory symptoms coupled with inflammatory changes on chest imaging after exclusion of infection and tumor progression. The incidence of all-grade CIP in clinical trials was estimated at 3%–5% with up to 70%–80% of cases responsive to glucocorticoid therapy.^5^ Patients who do not show improvement at 48–72 hours are typically treated with further immunosuppressive medications, such as infliximab, mycophenolate mofetil, intravenous immunoglobulins, or cyclophosphamide.^6^ Here, we present a case of a patient with melanoma with symptomatic and reversible diffuse pneumonitis associated with acute coronavirus HKU1 infection within days following the initiation of nivolumab and ipilimumab immunotherapy.

## Case presentation

A 65-year-old Caucasian man was diagnosed in February 2017 with a stage IVD BRAF wild-type cutaneous melanoma of the scalp with six intracranial metastases, innumerable bilateral lung metastases, and a peritoneal metastasis. He underwent bilateral craniotomies for excision of left temporal and right frontal lobe lesions with pathology showing melanoma with spindle cell and clear cell features. The day after corticosteroids were weaned off, combination nivolumab 1 mg/kg and ipilimumab 3 mg/kg was initiated.

**Figure 1:**
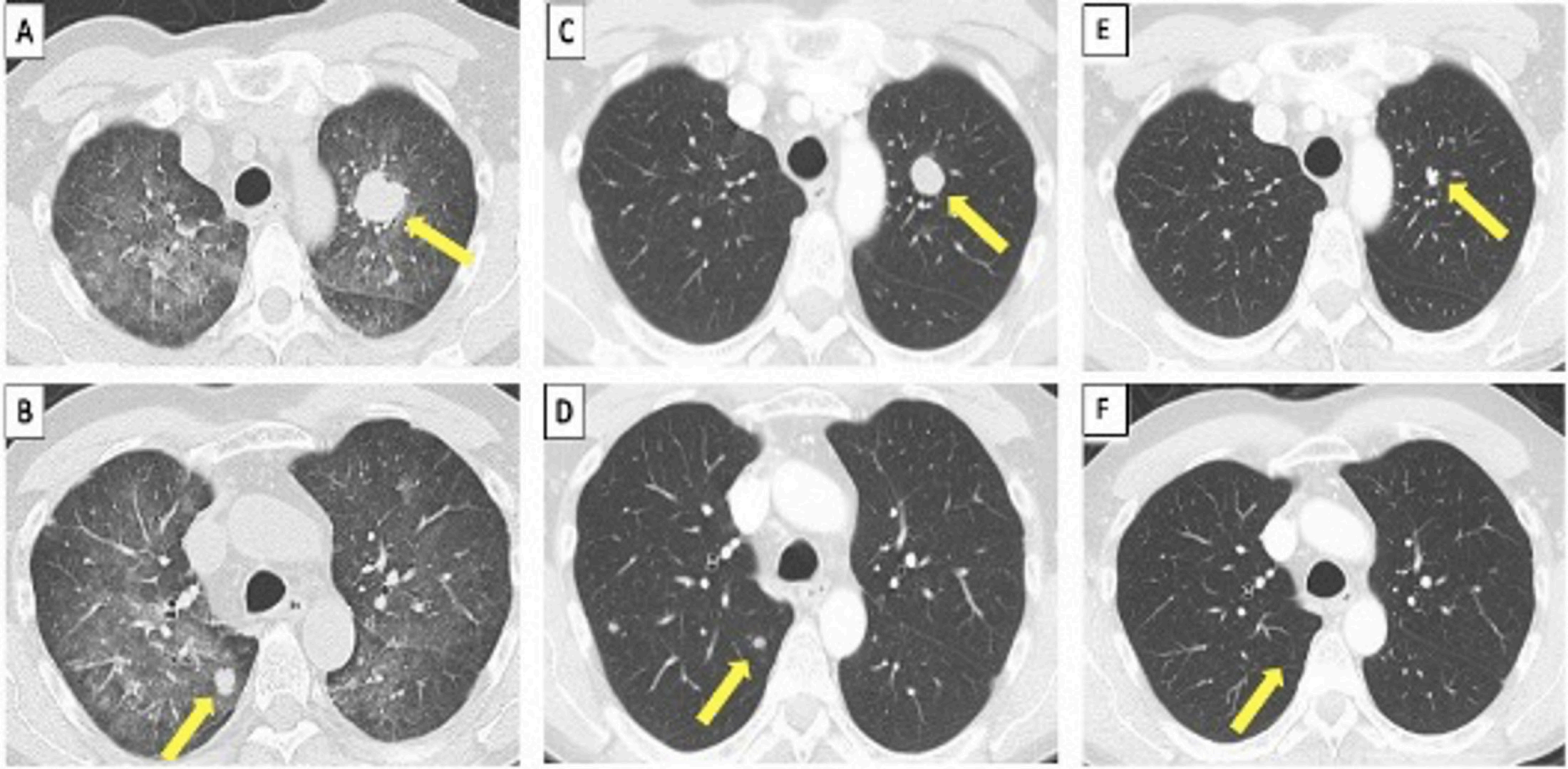
Comparison of the appearance of pulmonary metastasis and diffuse pneumonitis on CT scans. (A, B) In April 2017, multiple bilateral pulmonary metastases with superimposed ground glass opacities in the mid and upper lung fields. (C, D) In May 2017, resolution of diffuse pneumonitis and partial regression of lung nodules. (E, F) In February 2020, near-complete resolution of lung nodules.

In April 2017, 2 days after the first dose of nivolumab and ipilimumab, he developed cough productive of yellow sputum and dyspnea that persisted over the next 5 days. One week into ICI therapy, physical examination was notable for bilateral upper lung crackles without fever, hypotension, tachycardia, or hypoxia on room air. CT of the chest confirmed known pulmonary metastases superimposed by new diffuse ground glass opacification with slight central and upper lobe predominance (figure 1A, B). On hospital day 2, evaluation of respiratory viral pathogens with nasopharyngeal swab revealed the presence of coronavirus HKU1 (non-COVID-19). Complete blood count showed white cell count (WCC) 7.2 (10^9^/L), hemoglobin 12.9 (g/L), and platelets 252 (10^9^). Blood and sputum cultures revealed no growth and normal respiratory flora, respectively. The patient was initially diagnosed with CIP and treated with high-dose corticosteroids. Due to the patient’s rapid symptomatic benefit and our inability to exclude a role for the ICIs in exacerbating the newly diagnosed coronavirus infection, steroids were tapered off over a week rather than immediately discontinued.

In May 2017, a follow-up chest CT demonstrated resolution of ground glass opacification (figure 1C, D) at which time nivolumab 3 mg/kg monotherapy was initiated and continued for 25 doses until April 2018 without recurrence of pneumonitis. In April 2018, brain MRI showed postsurgical changes without evidence of metastases and chest and abdominal CT scans showed interval additional decrease in size and number of pulmonary nodules and right peritoneal nodule (figure 1E, F). 18F-fluorodeoxyglucose (FDG) positron emission tomography/CT scan showed no evidence of FDG-avid disease, supporting the likelihood of a metabolic complete systemic and cerebral response. Nivolumab monotherapy was discontinued after informed discussion of the known risks and benefits of both continuing and stopping therapy.

He was followed with clinical evaluation, brain MRI and torso CT scans every 3 months. At most recent follow-up in February 2020, 3 years after initial diagnosis and nearly 2 years of treatment he remains free of disease progression.

## Discussion and conclusions

CIP is uncommon; however, it can be life-threatening, necessitating early diagnosis and prompt intervention. This case study provides the first description of symptomatic pneumonitis in association with coronavirus HKU1 after combined anti-CTLA and anti-PD-1 blockade with ipilimumab and nivolumab in a patient with metastatic melanoma.

Coronavirus HKU1 was first discovered as a pathogenic cause for community-acquired pneumonia in 2005.^7^ A majority of patients experienced a monophasic infection leading to rapid recovery with median duration of hospitalization of only 5.5 days.^8^ Despite many patients having relatively mild infections, a minority of patients with comorbidities including malignancy, diabetes, and cardiovascular disease experienced severe infections leading to death. These patients died from early pneumonitis within approximately 1 week of hospital admission and there were no reports of late hyperinflammatory syndromes. Although there is a paucity of evidence for the use of corticosteroids in coronavirus HKU1 infections, steroids have been used to mitigate the secondary inflammatory response in severe coronavirus pneumonias caused by severe acute respiratory syndrome coronavirus (SARS-CoV) and Middle East respiratory syndrome coronavirus (MERS-CoV).^9 10^ Several retrospective studies and meta-analyses have consistently shown no clinical benefit from corticosteroid treatment and suggest impaired viral clearance of SARS-CoV and MERS-CoV.^10 11^ Despite the lack of benefit in phylogenetically similar coronavirus infections, this patient’s unique presentation of severe coronavirus HKU1 shortly after ICI therapy, and his clinical response to an initial corticosteroid dose, raised the possibility of synergistic pulmonary toxicity and led the authors to continue a short-course corticosteroids following viral detection.

Pulmonary adverse events of ICIs remain a complex diagnosis of exclusion. In a previous report, the median time to onset of pulmonary irAEs following ICI therapy was approximately 2.8 months (range: 0.3 to 19.2 months).^5^ Among patients treated with oral steroids, the median starting dose was prednisone 50 mg daily (range 20 to 80 mg) and median duration of treatment was 68 days (range 2 to 154 days). A number of factors distinguish our patient’s case from the typical ICI-related pneumonitis. First, the short interval of 1 week between dosing of ipilimumab and nivolumab and the occurrence of diffuse pneumonitis suggests an exacerbated immune response to a viral infection rather than a reaction solely to the ICI therapy. Furthermore, the rapid improvement in symptoms and radiographic pneumonitis with prompt short-course steroids is distinct from the usual case of ICI pneumonitis. Lastly, the absence of recurrent pneumonitis after re-challenging with anti-PD1 monotherapy supports the viral infection an inciting factor. Remarkably, the patient experienced a metabolic complete systemic and cerebral response to therapy that has been sustained for 3 years including 2 years after stopping therapy.

The novel COVID-19 has been confirmed in over 3 500 000 patients worldwide as of May 6, 2020 with a mortality rate of approximately 6.8%.^12^ In contrast to the monophasic coronavirus HKU1 and similar to SARS-CoV and MERS-CoV infections, COVID-19 has demonstrated a biphasic course. A recent cluster of 41 patients with SARS-CoV-2 infection demonstrated initial symptoms of fever (98%), cough (76%), and fatigue (44%) followed by development of dyspnea (55%) at a median 8 days, acute respiratory distress syndrome (27%) at median 9 days, and intensive care unit admission (39%) at median 10.5 days.^13^ Additionally, a recent retrospective study of 150 patients with confirmed COVID-19 discovered significantly elevated inflammatory markers in 68 deceased patients compared with 82 survivors with elevations in WCC (mean 10.6 vs 6.7; p<0.001), C reactive protein (mean 126.6 ng/mL vs 34.1 ng/mL; p<0.001), serum ferritin (mean 1297.6 ng/mL vs 614 ng/mL; p<0.001), and interleukin 6 (IL-6; mean 11.4 ng/mL vs 6.8 ng/mL).^14^ Together these clinical and laboratory findings suggest that COVID-19 mortality may be due to a late-phase virus-induced hyperinflammatory syndrome, rather than the direct effects of the virus itself and that this syndrome could be potentially exacerbated by ICI administration.

Several immune-modulating strategies have shown anecdotal efficacy for severe COVID-19 including biological therapies targeting inflammatory pathways with anti-IL-6 antibodies tocilizumab and sarilumab.^15^ Tocilizumab is a humanized monoclonal antibody to the IL-6 receptor that is Food and Drug Administration (FDA) approved for the management of cytokine release syndrome (CRS) for patients receiving chimeric antigen receptor (CAR) T-cell therapy and used for steroid-refractory irAEs from ICI therapy.^16 17^ The FDA has recently approved COVACTA, a randomized, double-blind, placebo-controlled phase III trial of tocilizumab plus standard of care (SOC) compared with placebo plus SOC.^18^ A trial of the anti-IL-6-receptor sarilumab for patients with severe COVID-19 infections is also ongoing.^19^ Due to the limited access to such specific anticytokine agents, many institutions are substituting high-dose steroids for patients with elevated inflammatory marker profiles with anecdotal evidence of benefit (Charles Drake, personal communication, 2020). Given this pathophysiology, corticosteroid use might be particularly appropriate for patients with COVID-19 pneumonitis in the setting of ICI therapy.

This case report highlights a potential interaction between coronavirus infection and ICI therapy that could lead to exacerbation of pneumonitis. In patients with suspected CIP, several irAE guidelines recommend evaluation of alternative etiologies including nasopharyngeal testing for viral infections, which may facilitate more accurate diagnosis and appropriate therapy.^20–22^ While this case was related to coronavirus HKU1 and was readily treated, it highlights a potential concern regarding the impact of ICI therapy on the effects of both common coronaviruses and the more virulent SARS-CoV-2 infection. Considering the prevalence COVID-19 worldwide, we recommend SARS-CoV-2 testing for patients on ICIs with symptoms (fever, dyspnea, or cough) regardless of the presence or absence of infiltrates on imaging. In patients under investigation for COVID-19, we further recommend that ICI treatment should be withheld until SARS-CoV-2 infection is excluded. Due to the potential risk of exacerbating an asymptomatic infection, we also recommend that for patients with a potential exposure to a person with COVID-19, ICI therapy be withheld until SARS-CoV-2 infection can be ruled out.

The case sheds some light on the potential biology of the lethal pulmonary toxicity associated COVID-19. The elevated inflammatory markers seen in severe SARS-CoV-2 infections are reminiscent of a low level of the CRS seen with CAR T-cell therapy, a condition where selective immunosuppressive treatment can be effective. These observations support the ongoing study of immunosuppressive therapies including high-dose corticosteroids and anti-IL-6 receptor antibodies in patients with severe COVID-19.
